# Stromal androgen receptor regulates the composition of the microenvironment to influence prostate cancer outcome

**DOI:** 10.18632/oncotarget.3873

**Published:** 2015-04-19

**Authors:** Damien A. Leach, Eleanor F. Need, Roxanne Toivanen, Andrew P. Trotta, Helen M. Palenthorpe, David J. Tamblyn, Tina Kopsaftis, Georgina M. England, Eric Smith, Paul A. Drew, Carole B. Pinnock, Peng Lee, Jeff Holst, Gail P. Risbridger, Samarth Chopra, Donald B. DeFranco, Renea A. Taylor, Grant Buchanan

**Affiliations:** ^1^ The Basil Hetzel Institute for Translational Health Research, University of Adelaide, SA, Australia; ^2^ Department of Anatomy and Development, Monash University, VIC, Australia; ^3^ Urology Unit, Repatriation General Hospital, SA, Australia; ^4^ Department of Surgical Pathology, SA Pathology at Flinders Medical Centre, Bedford Park, SA, Australia; ^5^ School of Nursing and Midwifery, Flinders University, Bedford Park, SA, Australia; ^6^ Department of Pathology and Urology, New York University, NY, USA; ^7^ Origins of Cancer Laboratory, Centenary Institute, NSW, Australia; ^8^ Sydney Medical School, University of Sydney, NSW, Australia; ^9^ Department of Urology, St Vincent's Hospital, Sydney and Garvan Institute; ^10^ Department of Pharmacology and Chemical Biology, University of Pittsburgh, PA, USA; ^11^ Department of Physiology, Monash University, VIC, Australia

**Keywords:** prostate cancer, androgen receptor, stroma, fibroblasts, extracellular matrix

## Abstract

Androgen receptor (AR) signaling in stromal cells is important in prostate cancer, yet the mechanisms underpinning stromal AR contribution to disease development and progression remain unclear. Using patient-matched benign and malignant prostate samples, we show a significant association between low AR levels in cancer associated stroma and increased prostate cancer-related death at one, three and five years post-diganosis, and in tissue recombination models with primary prostate cancer cells that low stromal AR decreases castration-induced apoptosis. AR-regulation was found to be different in primary human fibroblasts isolated from adjacent to cancerous and non-cancerous prostate epithelia, and to represent altered activation of myofibroblast pathways involved in cell cycle, adhesion, migration, and the extracellular matrix (ECM). Without AR signaling, the fibroblast-derived ECM loses the capacity to promote attachment of both myofibroblasts and cancer cells, is less able to prevent cell-matrix disruption, and is less likely to impede cancer cell invasion. AR signaling in prostate cancer stroma appears therefore to alter patient outcome by maintaining an ECM microenvironment inhibitory to cancer cell invasion. This paper provides comprehensive insight into AR signaling in the non-epithelial prostate microenvironment, and a resource from which the prognostic and therapeutic implications of stromal AR levels can be further explored.

## INTRODUCTION

Prostate cancer causes more than 28,000 deaths each year in the United States [1]. Critically, 10-33% of clinically localized cancers treated by surgery will eventually progress, indicative of undetected pre-existing metastatic disease [2, 3]. Although epithelial differentiation scored by Gleason pathology at diagnosis aids in prognosis and management, it is imprecise in prediction of sub-clinical metastases or low grade tumors at risk of rapid progression. Recent studies of various solid tumors suggest that the stromal microenvironment may yield additional diagnostic information and novel avenues for therapeutic intervention [4-7].

Prostate development and homeostasis requires bidirectional signaling between epithelial cells and stromal constituents, including fibroblast and smooth muscle cells, vasculature, soluble factors and extracellular matrix (ECM) proteins. This signaling is disrupted in cancer [8-10], where the stroma becomes disorganized, normal non-malignant prostatic fibroblasts (NPFs) are replaced by activated cancer-associated fibroblasts (CAFs), and the composition of the ECM is altered [11-14]. Compared to NPFs, CAFs exhibit increased proliferation and migratory behavior [15], induce malignancy in non-tumorigenic prostate epithelial cells [16-18], and provoke tumor progression via secretion of signaling factors [19-22]. Moreover, genomic-level studies have identified prognostic CAF-specific gene signatures in digestive, non-small cell lung, breast and prostate cancers [4, 23-25].

In the adult prostate, activation of epithelial androgen receptor (AR) by testosterone (T) and 5α-dihydrotestosterone (DHT) is necessary for cell survival and regulation of seminal fluid proteins including prostate specific antigen (PSA) [26], which is used clinically for tumour detection and monitoring. Although targeting androgens through ablation is therefore an effective initial treatment strategy for advanced cancer, most reoccur by refractory reactivation of epithelial AR [27-29]. In prostate development however, it is the stromal AR that is necessary for establishment of normal prostatic architecture, and for epithelial differentiation and function [30]. Decreased stromal AR expression in cancer has been associated with tumor resistance to androgen deprivation [31], and with relapse and progression following radical prostatectomy [25, 32, 33]. Currently however, we do not know how decreased stromal AR contributes to prostate cancer progression, or indeed how androgen action differs between prostate stromal and epithelial cells.

In this study, we compared AR levels in epithelial and stromal compartments of patient-matched benign and malignant prostate tissue, and demonstrate an association between low stromal AR levels and death from prostate cancer at one, three and five years post diagnosis. This is the first time that stromal AR changes have been shown to be specific to the immediate cancer microenvironment and not due to differences between patients, and are related to adjacent malignant but not benign regions of the same prostate. We further show that androgen signaling in human prostatic myofibroblasts induces a microenvironment inhibitory to the movement and invasion of tumor cells, primarily by altering ECM composition. This protective AR-mediated phenotype in prostate cancer-associated stroma has implications for understanding the early stages of cancer progression, and for the use of androgen withdrawal in the absence of surgical management.

## RESULTS

### Association of AR levels in epithelium and stroma of benign and malignant prostate tissue with clinical parameters

**Figure 1:**
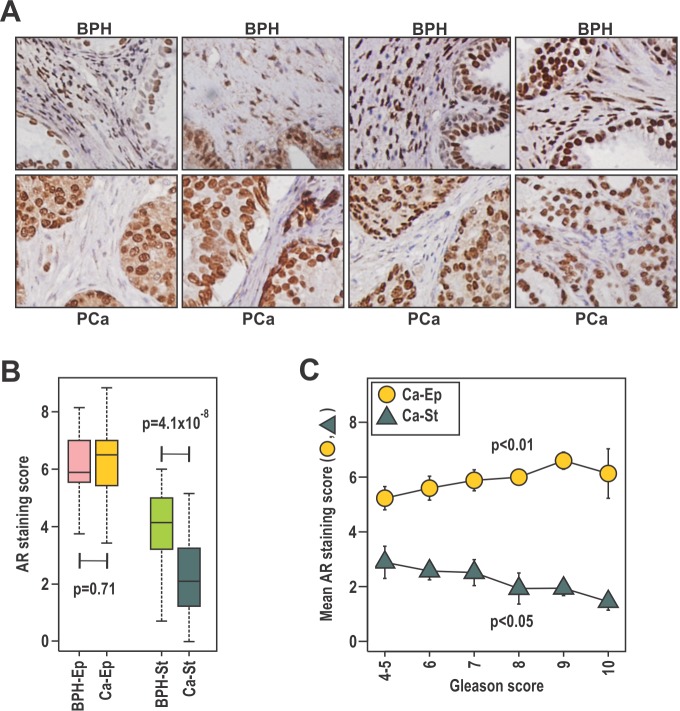
The expression of stromal AR is related to clinical parameters and outcomes of prostate cancer **A**-**C**. Patient-matched duplicate cores of BPH and cancer were immunostained with anti-AR antibody. Samples were scored by two independent researchers, using a scale of high (3), moderate (2), low (1) intensity or absent (0) immunostaining in the epithelia and stroma and averaged between the duplicate samples and scorers. **B**. Scores were evaluated in relation to disease state for stroma (St) and epithelia (Ep) and compared using the Wilcoxon Rank-Sum test **C**. Mean AR score ± SEM for both the cancer stroma (Ca-St) and epithelia (Ca-Ep) was calculated for each Gleason grade.

The relationship between prostate cancer outcome and AR levels in stroma and epithelium was investigated by AR immunohistochemistry on 64 patient-matched BPH and prostate cancer samples in patients of median age 87 years (Fig. 1A). Similar to a previous report [33], the median intensity of AR staining was lower in stroma than in epithelia (Fig 1A, B). Median AR levels were similar in malignant and benign epithelia, but were lower in cancer-associated compared to benign stroma (p=4.1 × 10^−8^, Fig. 1B, Table 1A). Consistent with established clinical associations, patients with higher Gleason score had a greater extent of disease, higher serum PSA levels, and were more likely to have died from their disease at censure. Additionally, a positive association between serum PSA levels was observed for AR content in cancer epithelia (p=0.004), but not with the other AR measures (Supplementary Fig. S1A-D). Higher Gleason score was associated with a higher median AR level in cancer epithelia (p<0.05) and lower AR in cancer-associated stroma (p<0.05; Fig.1C, Table 1A). Previous studies have reported an association between low stromal AR levels and biochemical recurrence [25, 32-34]. Here we assessed stromal and epithelial AR levels in paired BPH and cancer samples from the same patients, allowing discrimination of changes specific to cancer stroma from those related to an individual patient or prostate. Critically, we observed that low AR levels in cancer stroma, but not BPH stroma, were associated with prostate cancer related death (p=0.02; Table 1A) at censure, which was a minimum five years post initial diagnosis. The level of AR in cancer or BPH epithelia was not associated with outcome. We next dichotomized the cohort by median AR level in cancer epithelia or cancer stroma. High epithelial AR levels was associated with the extent of disease, Gleason score and serum PSA (p<0.05), but not with outcome (Table 1B). Conversely, low AR in cancer stroma was associated with more extensive disease, and a greater risk of prostate cancer-related death (p<0.05, Table 1B). At the time of censure, the median prostate cancer specific survival for patients with low stromal AR was 622 days, which was significantly less than patients with high stromal AR expression at 2528 days (p=0.013). Finally, we observed lower 1, 3, and 5 year prostate cancer specific survival in patients with low stromal AR (30% at 5 years) compared to high stromal AR (56% at 5 years; Table 1B). Despite AR in epithelial cells being more related to clinical parameters of histologically aggressive disease, our data suggest the intriguing possibility that AR in fibroblasts plays a more critical role in protecting against prostate cancer progression. Moreover, AR level in BPH stroma from the same patients was not associated with progression, supporting the existence of pathological cancer associated stroma in prostate cancer.

**Table 1:** AR levels in epithelia and stroma of prostate cancer and patient-matched benign regions

A.		all (n=64)^@^	Gleson <=7 (n=24)^%^	Gleason >7 (n=39)	p value^#^	PCa Death NO=(n=38)^%^	YES (n=26)	p value^#^
**age**		87 (60-100)	86 (67-97)	88 (60-100)	ns	86 (67-98)	88 (60-100)	ns
**% Prostate cancer**		50 (10-100)	**22 (10-88)**	**80 (10-100)**	**<0.0001**	**30 (10-100)**	**78 (12-99)**	**0.0051**
**Gleason score**		8 (4-10)				**7 (4-10)**	**9 (7-10)**	**0.0002**
**PSA (ng/ml)**		16.5 (0.5-8300)	**6 (0.5-174)**	**26 (1-8300)**	**0.0011**	14.3 (0.5-174)	18.4 (0.9-8300)	ns
**PCa death**		26	**3**	**23**	**0.0001**^&^			
**PCa-epithelia**	**AR score**	6.50 (0.67-8.83)	**5.57 (3.43-7.57)**	**6.36 (0.67-8.83)**	**0.0179**	6.50 (3.42-8.14)	6.36 (0.67-8.83)	ns
**PCa-stroma**		2.10 (0-5.15)	**2.67 (0-4.86)**	**1.71 (0.07-5.13)**	**0.0262**	**2.21 (0-4.86)**	**1.33 (0.21-5.15)**	**0.028**
**BPH-epithelia**		5.89 (3.17-8.14)	6.33 (3.75-8.14)	5.86 (3.17-7.4)	ns	6.30 (3.17-8.14)	5.86 (3.75-7.40)	ns
**BPH-stroma**		4.14 (0.71-6.00)	**4.75 (2.27-6)**	**3.77 (0.71-5.57)**	**0.0155**	4.00 (0.71-6.00)	4.5 (1.07-5.57)	ns

B.		all (n=64)^@^	AR Low PCa-Ep * (n=28) 5.43 (0.67-6.36)	AR High PCa-Ep (n=29) 7.00 (6.50-8.83)	p value^#^	AR Low PCa-St * (n=29) 1.23 (0.00-2.10)	AR High PCa-St (n=28) 3.28 (2.14-5.15)	p value^#^
**age**		87 (60-100)	**88 (71-100)**	**84 (60-94)**	**0.0115**	88 (71-100)	85 (60-95)	ns
**% Prostate cancer**		50 (10-100)	**25 (10-100)**	**80 (10-100)**	**0.0021**	80 (12-100)	33 (10-99)	0.046
**Gleason score**		8 (4-10)	**7 (4-10)**	**9 (6-10)**	**0.0139**	9 (5-10)	7 (4-10)	ns
**PSA (ng/ml)**		16.5 (0.5-8300)	**8 (0.5-174)**	**25 (2-8300)**	**0.0161**	17 (1-2617)	16 (1-8300)	ns
**PCa death**		24	13	11	ns^&^	**16**	**8**	**0.0245**^&^
**PCa-epithelia**	**AR score**	6.50 (0.67-8.83)				6.50 (0.67-8.83)	6.36 (3.34-7.69)	ns
**PCa-stroma**		2.10 (0-5.15)	2.07 (0-5.15)	2.10 (0.07-4.86)	ns			
**BPH-epithelia**		5.89 (3.17-8.14)	5.86 (3.75-8.00)	6.15 (3.75-8.14)	ns	5.89 (3.75-7.43)	5.86 (3.86-8.14)	ns
**BPH-stroma**		4.14 (0.71-6.00)	4.50-1.50-6.00)	3.42 (0.71-5.79)	ns	4.17 (0.71-6.00)	4.00 (1.17-5.71)	ns
**PCa specific survival**		1-yr survival rate				88%	65%	
		3-yr survival rate				68%	45%	
		5-yr survival rate				56%	30%	

@ Data for age, percent cancer in sample (% prostate cancer), Gleason score, PSA and AR staining score are presented as median (range), and for prostate cancer related death as absolute numbers

% Gleason score and Prostate cancer (PCa) death status available at censure for 63/64 patients

* Samples dichotomized about the median AR score

# Two-tailed Mann-Whitney U test unless otherwise indicated

& Barnard's Exact test

### Myofibroblast AR expression modulates patient cancer cell response to castration in a tissue recombination model

**Figure 2:**
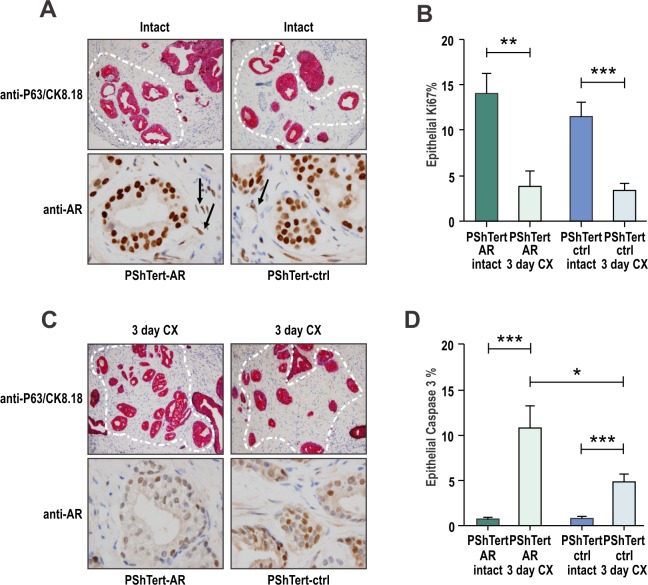
Loss of myofibroblast AR protects cancerous prostatic epithelia from castration induced apoptosis Tissue recombination of patient prostate cancer tissues co-grafted with either PShTert-AR or PShTert-ctrl myofibroblasts into immune-deficient host mice. After 8 weeks, host mice were castrated for a further three days. **A**. Human tissue was identified by dual immunostaining of basal cell marker p63 (brown stain) and epithelial marker CK8/18 (pink stain); cancer foci were p63^−^CK8/18^+^ *highlighted by white outline*. AR levels were assessed in samples immunostained with anti-AR antibody. **B**. Epithelial proliferation was determined by the percentage of cells immunostained for anti-Ki-67. **C**. Human cancer tissue grafts from castrated mice was assessed for CK8/18, p63 and AR as described in (A). **D**. Epithelial cell death was measured through cleaved caspase-3 immunostaining and percent positive cells counted (*, p<0.05, **, p<0.01, ***, p<0.001, Student's T-test).

To investigate the role of stromal AR in cancer, we utilized *in vivo* tissue recombination [35]. Human prostate cancer tissues obtained from four patients with moderate (Gleason 7) tumors were combined as heterotypic recombinants with AR positive human prostate PShTert-AR myofibroblasts or AR negative PShTert-ctrl and sub-renally grafted into immunodeficient NOD-SCID mice. Human cancer cells combined with both PShTert-AR and PShTert-controls formed phenotypically similar ductal structures that stained positive for the human-specific epithelial marker p63/CK8.18 (Fig. 2A). The survival of cancer foci, detected as p63^−^/CK8.18^+^, was similar in grafts from the four patients with PShTert-AR (65%, 11/17) and PShTert-ctrl (56%, 13/23) lines. As expected, a significantly lower proportion of stroma in the grafts containing PShTert-ctrl myofibroblasts expressed AR (p<0.01; Supplementary Fig. 1E), with residual stromal AR expression arising from mouse-derived stroma. Castration resulted in significantly reduced tumor cell proliferation in both PShTert-AR (p<0.01; Fig. 2B) and PShTert-ctrl myofibroblast (p<0.001; Fig. 2B) grafts, a reduction in cancer p63^−^/CK8.18^+^ foci (Fig. 2C), and a higher percentage of apoptotic cancer cells (caspase-3 positive; p<0.001; Fig. 2D). More importantly, there was significantly less cancer cell apoptosis in grafts with PShTert-ctrl cells in comparison to grafts with PShTert-AR cells (p<0.05; Fig. 2D). This latter result suggests that low stromal AR reduces apoptosis of primary cancer cells in response to androgen deprivation *in vivo*.

### Transcription activity, gene regulation, chromatin targeting and proliferation of prostate epithelial and myofibroblast cells diverge in response to androgens

**Figure 3:**
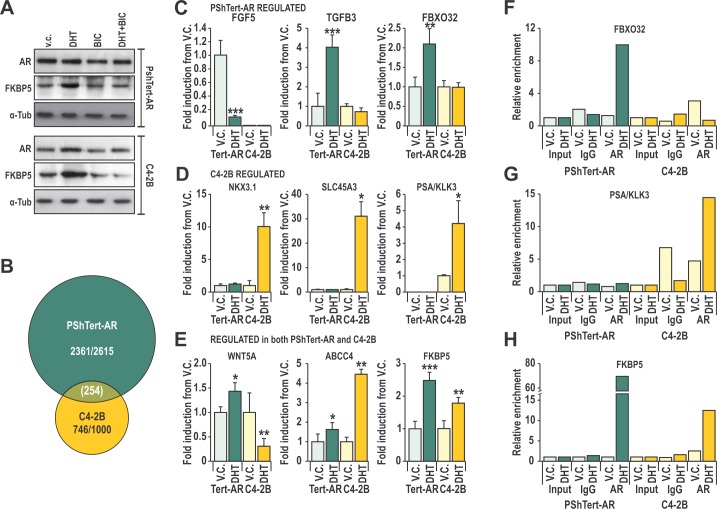
Cell specificity of AR action may be mediated by interactions of AR with DNA **A**. Lysates from C4-2B and PShTert-AR cells treated with or without 10 nM DHT and 10 μM bicalutamide (BIC) were probed for AR and FKBP5. **B**. Affymetrix 1.0st Gene Array of 10 nM DHT or vehicle control (V.C.) treated PShTert-AR or C4-2B cells, presented as a Venn-diagram of genes with >0.5 log_2_ fold change in expression between treatments. **C**-**E**. Microarray was validated via RT-qPCR of independent samples produced under the same conditions. Data is represented as mean + SEM of triplicate biological replicates (V.C. vs DHT * p<0.05, ** p<0.01, ***p<0.001 Student's T-test). **F**-**H**. Chromatin immunoprecipitation (ChIP) was performed on C4-2B and PShTert-AR cells treated with 10 nM DHT or vehicle, and immunoprecipitated with anti-AR N20 or nonspecific IgG antibody. ChIP samples were quantified by RT-qPCR and mean percent input for each binding region in the proximity of (**F**) FBXO32, (**G**) PSA and (**H**) FKBP5 was normalized to a non-specific binding region.

We next sought to define the molecular actions of AR in PShTert-AR myofibroblasts, and to contrast those from androgen responses of prostate cancer epithelial C4-2B cells. These lines have a comparable levels of AR protein (Fig. 3A), and both have a functional AR signaling pathway as demonstrated by increased FKBP5 protein levels and probasin reporter (PB3) transactivation in response to DHT (Fig. 3A, Supplementary Fig. S2A). These responses are AR specific, and could be blocked by the AR antagonist, BIC (Fig. 3A, Supplementary Fig. S2A). Transcriptional reporter assays suggest however, that the DHT response of AR is 10-fold less sensitive in myofibroblasts than in epithelia (Supplementary Fig. S2B), and is not due to technical limitations such as reporter level (Supplementary Fig. S2C). Furthermore, only classical androgen agonists (DHT and T) and medroxyprogesterone acetate (MPA) could produce a transcriptional response in PShTert-AR cells (Supplementary Fig. S2D), compared with the expected broader ligand responses in C4-2B cells (Supplementary Fig. S2E). Nevertheless, the ability of the AR to stimulate a panel of AR-targeted reporters was consistent between PShTert-AR and C4-2B cells (Supplementary Fig. S2F).

**Figure 4:**
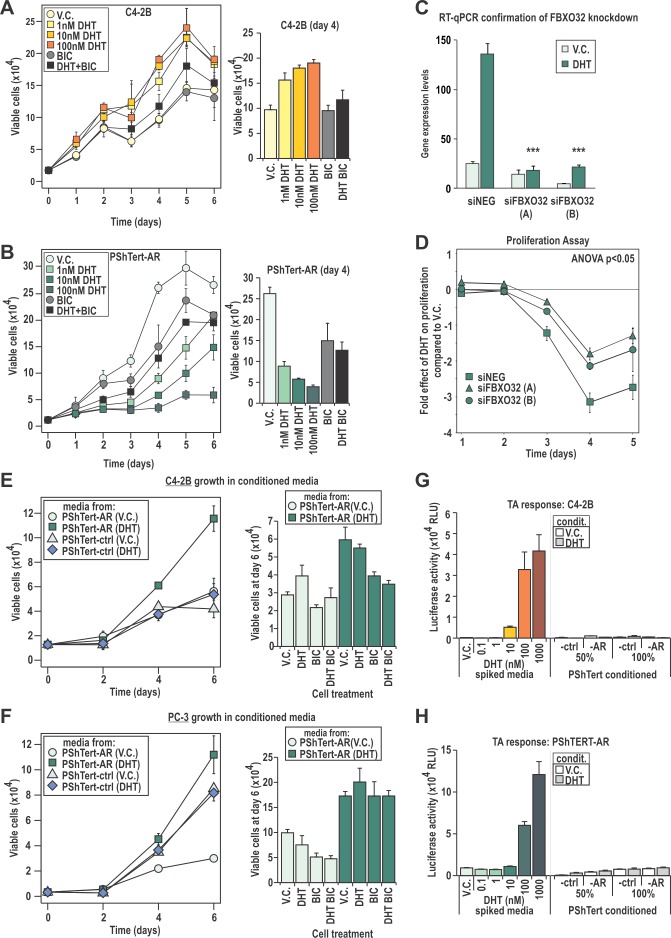
C4-2B and PShTert-AR cells have different proliferative responses to DHT **A**-**B**. Proliferative response of C4-2B and PShTert-AR cells to 10 nM DHT was measured daily via Trypan blue exclusion assays. **C**,**D**. The androgen mediated gene and DNA-licensing factor, FBXO32, was silenced via siRNA (**C**) and the effect on PShTert-AR growth in response to 10 nM DHT was measured via Trypan blue exclusion assay (**D**). **E**,**F**. The effect of conditioned media from PShTert-AR and PShTert-ctrl on C4-2B and PC-3 cells was measured as in **A**. Data represents the mean number of viable cells in triplicate wells ± SEM. **G**,**H**. The presence of DHT in the conditioned media was assessed via transactivation assays performed on C4-2B (**G**) and PShTert-AR (**H**) cells. Data presented as mean relative light units (RLU) ± SEM of six independently transfected wells.

In order to more precisely define the transcriptional role for AR in PShTert-AR cells, we performed expression microarray analysis, identifying 2615 DHT regulated genes in PShTert-AR myofibroblasts and 1000 in C4-2B epithelial cells (>0.5 log2 fold change). Importantly, only 254 of those regulated genes were common between the two cell lines, and half of those (127/254) were regulated in the opposite direction (Fig. 3B). RT-qPCR analysis of an independent sample set confirmed the uniquely regulated (Fig 3C-D) and similarly regulated (Fig. 3E) responses to DHT in each cell line. The AR-specific nature of myofibroblast responses was confirmed by their absence in PShTert-ctrl cells (Supplementary Fig. S3). ChIP analysis of well-characterized androgen target genes suggests that divergent AR occupancy of promoters/enhancers is responsible for the cell-specific regulation by DHT (Fig. 3F-H), consistent with a contemporary understanding of AR chromatin targeting [36]. We next applied pathway analysis to the top 1000 regulated genes in each cell line, which in PShTert-AR cells comprised 390 upregulated and 610 downregulated genes, and in C4-2B cells 648 upregulated and 352 downregulated genes. DHT-treated myofibroblasts were enriched in adhesion and ECM organization, but depleted in cell cycle and migration (Supplementary Table 2). In contrast, DHT in C4-2B cells drives processes of lipid and fatty acid synthesis and migration, and depletion of apoptosis (Supplementary Table 2). Importantly, a considerable number of pathways were regulated in opposite directions by DHT in epithelial and myofibroblast cells, despite limited commonality in regulated genes (Fig. 3B; Supplementary Table 2). Consistent with the divergent gene responses, DHT stimulated C4-2B cells to proliferate as previously reported [37] (p<0.05; Fig. 4A), but inhibited PShTert-AR growth in a dose-dependent manner (p<0.001, Fig. 4B). Cell death did not vary significantly between treatments in C4-2Bs over the 6 day period, but was significantly altered by all doses of DHT in PShTert-AR cells at days 3 and 4 (p<0.05; 5-20% of viable cells; Supplementary Fig. S4). Importantly, BIC reversed these effects, confirming AR mediation of the divergent growth responses (Fig. 4A, B; *right panels*).

One mediator of the anti-proliferative effect of androgen in myofibroblasts may be the fibroblast-specific androgen regulated *F-box protein 32* (*FBXO32*) gene product. FBXO32 is a member of the family of DNA-licensing proteins that regulates progression from G1 phase by inhibiting cyclin D1 [38]. To determine whether FBXO32 could regulate proliferation in AR expressing myofibroblasts, we used siRNA knockdown (Fig. 4C). FBXO32 depletion partially reversed the inhibitory effect of DHT on myofibroblast cell growth over the course of a five day period (p<0.05; Fig. 4D). Together, the above results demonstrate that AR in epithelial and myofibroblast lineages plays distinct roles, one of which is to direct divergent proliferative responses to DHT.

### AR action in myofibroblasts promotes epithelial cancer proliferation

We next considered whether AR activity in myofibroblasts could affect epithelial growth. Conditioned media was collected from PShTert-AR and PShTert-ctrl myofibroblasts treated with or without DHT. Compared to vehicle, media from DHT treated AR positive myofibroblasts increased C4-2B and PC-3 proliferation by 1.64 and 2.72 fold respectively (p<0.05, Fig. 4E, F). Media from DHT treated AR negative myofibroblasts did not alter the proliferative response of either epithelial line. The addition of DHT to vehicle conditioned media from PShTert-AR cells enhanced proliferation of C4-2B but not AR negative PC-3 cells, an effect reversed by co-treatment with BIC (Fig. 4E, F). In contrast, DHT supplementation had no effect on the higher proliferation seen with DHT stimulated myofibroblast conditioned media (Fig. 4E, F). Residual DHT from the conditioning process was not responsible for these effects, as high-sensitivity transcriptional reporter assays did not reveal any androgen activity in conditioned media (Fig. 4G, H). It appears likely from these studies that DHT stimulation of AR positive myofibroblasts produces secreted, soluble factors that are pro-proliferative to epithelial cells.

### AR action in prostate myofibroblast cells controls adherence of myofibroblast cells

**Figure 5:**
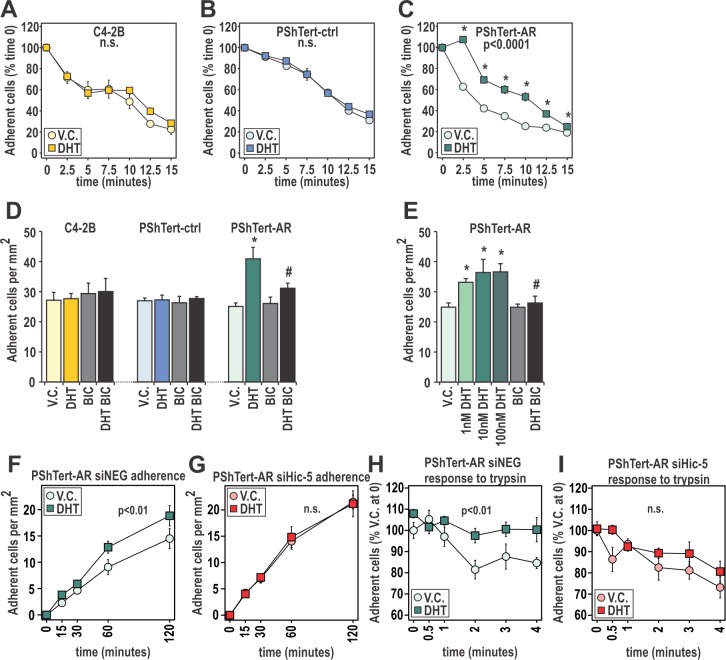
DHT has pro-adherent effects on fast and long term adherence of myofibroblast cells **A**-**C**. The quantity of C4-2B, PShTert-ctrl, and PShTert-AR cells, treated with 10 nM DHT or equivalent vehicle (V.C.), remaining after trypsinization over 15 min was measured using crystal violet staining. Presented as mean ± SEM of six technical replicates, and representative of three independent experiments. **D**,**E**. Adherence was measured by manually counting the number of 10 nM DHT, V.C. or 10 μM bicalutamide (BIC) treated C4-2B, PShTert-ctrl, and PShTert-AR cells adhering after 30 min. Data is presented as mean ± SEM of four samples and is representative of three independent experiments. (* p<0.05 V.C. vs DHT, # p<0.05 DHT vs DHT+BIC Student's T-test). **F**,**G**. PShTert-AR cells transfected with siRNA against Hic-5 or scrambled control were assayed for adherence as described in D but measured over a 2 h period. Data is presented as mean ± SEM of four replicates and representative of three independent experiments. **H**-**I**. Hic-5 contribution to androgen-mediated attachment was assayed as described in A-C. For all time course adherence data, significance (p<0.05) was determined by one-way ANOVA.

As pathways involving adhesion were highly enriched in DHT treated myofibroblasts, we next assessed whether this translated to altered attachment. Treatment with DHT had no effect on trypsinization of C4-2B cells or PShTert-ctrl cells, but increased retention of PShTert-AR cells by 25.1 ± 3.6% to 44.7 ± 1.8% (p<0.0001, Fig. 5A-C). This response was DHT dose dependent and reversible by BIC (p<0.05; Supplementary Fig. S5), thus demonstrating AR involvement. Furthermore, DHT treatment significantly increased attachment of PShTert-AR cells by 33-44% at 30 min in a dose-dependent manner, suggestive of an additional non-genomic effect (p<0.05, Fig. 5D, E). This response was measurable for 4 h and could be reversed by BIC (Fig. 5D), but did not occur with either C4-2B or PShTert-ctrl cells.

We recently reported that hydrogen peroxide-inducible gene 5 (Hic-5/*TGFB1I1*), a predominantly fibroblast-specific AR coregulator and a component of the focal adhesion (FA) complex, plays an important role in AR-mediated activity in myofibroblasts [39-41]. To assess whether Hic-5 might be involved in DHT/AR-mediated adherence, we utilized siRNA knockdown in PShTert-AR cells (Supplementary Fig. S6). Compared to negative siRNA control, depletion of Hic-5 abolished the effect of DHT on myofibroblast adherence (Fig. 5F, G). Similarly, Hic-5 knockdown eliminated the positive effect of DHT pretreatment on myofibroblast attachment (Fig. 5H, I). AR however retained the capacity to regulate FKBP5 expression when Hic-5 was depleted, implying that decreased adherence was not due to absolute loss of AR activity (Supplementary Fig. S6). Together, these results suggest an active role for AR in myofibroblast attachment, mediated via cellular interactions with a known AR coregulator.

### AR action in prostate myofibroblasts alters the ECM to increase cancer cell attachment and decrease cancer cell migration and invasion

**Figure 6:**
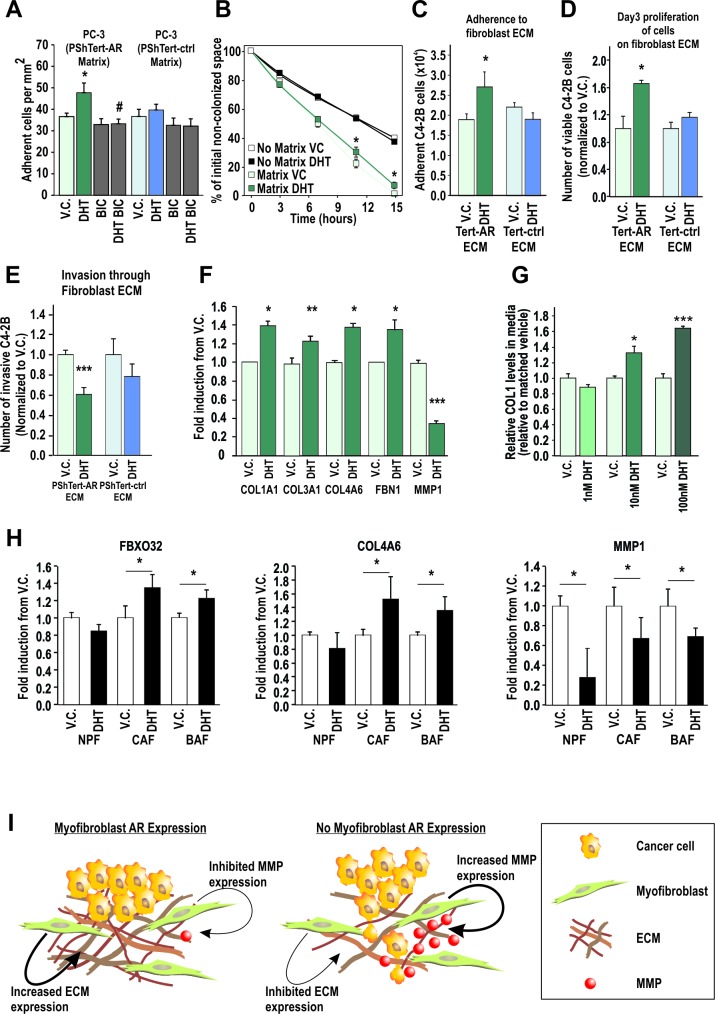
AR in cancer associated fibroblasts and model of AR action in prostate stroma **A**. PC-3 attachment to ECM deposited by PShTert-ctrl and PShTert-AR cells treated with or without 10 nM DHT ± 10 μM BIC was measured as described in Fig. 5D. Data presented as mean adherence per mm^2^ of four wells ± SEM. (* p<0.05 vehicle control (V.C.) vs DHT, # p<0.05 DHT vs DHT+BIC Student T-test). **B**. Migration of PC-3 cells over matrices created from V.C. or DHT treated PShTert-AR myofibroblasts was assessed by measuring the area of the cell-free gap over a 15 hour time period and calculated as a percentage of time point 0. Data represents mean ± SEM of three replicates. **C**-**E**. PShTert-AR or PShTert-ctrl cells were grown to confluence on a gelatin layer and allowed to deposit a 3D-ECM for 8 d following 10 nM DHT or V.C. treatment before myofibroblast removal. **C**. Adherence of 5 × 10^4^ C4-2B cells to the 3D-matrices was determined after an hour. Data is presented as mean ± SEM of four replicates and is representative of three independent experiments. **D**. The effect of the 3D-matrices on epithelial proliferation was determined via Trypan blue exclusion assay. Data is presented as mean ± SEM of four replicates and is representative of three independent experiments. **E**. Invasion of calcein-labeled C4-2B cells through the myofibroblast 3D-matrices was determined via a modified Boyden chamber technique. Data is presented as mean ± SEM of six samples and is representative of three independent experiments. **F**. RT-qPCR analysis for expression of selected ECM genes in PShTert-AR cells. Data represents the mean + SEM from triplicate biological replicates. **G**. ELISA analysis of collagen-1 (COL1) levels in conditioned media from DHT treated PShTert-AR cells. Data is presented as the mean + SEM from six replicates representative of two independent experiments. **H**. RT-qPCR gene analysis in human patient cancer associated fibroblasts (CAF), BPH associated fibroblasts (BAF), and normal prostatic fibroblasts (NPFs), isolated and treated with either V.C. or 100 nM DHT. Data represents the mean of technical triplicates (± SEM) from N=1 for each cell type (in all panels * p<0.05, ***p<0.001, Student's T-test). **I**. Model of AR action in prostate myofibroblasts. The AR signaling in myofibroblasts causes increased production of ECM components and inhibition of MMP enzymes. When AR signaling in myofibroblasts is lost, decreased expression of ECM components and enhanced MMP expression create an environment which decreases cancer cell attachment and increases cancer cell invasion.

As we had observed increased attachment and altered expression of ECM components with DHT treatment in the myofibroblast cells (Supplementary Table 2), we next measured adherence of epithelial cells to the myofibroblast-deposited matrix. PC-3 attachment to matrix generated by DHT treated PShTert-AR cells was increased 31 ± 12% over matrix from vehicle treated cells, and could be inhibited by BIC (p<0.05, Fig. 6A). In contrast, PC-3 adhesion to matrix from PShTert-ctrl cells was unaffected by ligand (Fig. 6A). Similarly, PC-3 migration over ECM generated by DHT treated PShTert-AR cells was significantly less than migration over ECM produced under vehicle control treatment after 7 (22±3% vs 30±3.5%) and 11 (1±1.3% vs 7±2.4%) hours (p<0.05, Fig 6B, Supplementary Fig. S7). As previously reported, cancer cell migration was significantly faster over ECM than cancer cell migration over plastic alone [42]. We next assessed the adherence of cancer cells to a myofibroblast conditioned 3D-ECM as previously described [43]. Consistent with the above results, a significant increase in C4-2B attachment (Fig. 6C) and proliferation (Fig. 6D) was only observed in gelatin conditioned by DHT-treated PShTert-AR cells, but not with gelatin conditioned by vehicle-treated PShTert-AR cells, or with vehicle- or DHT-treated PShTert-ctrl line (Fig. 6C, D). We also identified a significant decrease in invasion of the cancer cells through DHT-treated PShTert-AR gelatin matrix in comparison to matrix conditioned by vehicle treated PShTert-AR or DHT-treated PShTert-ctrl cells (Fig. 6E).

Candidate RT-qPCR analysis confirmed DHT upregulation of ECM proteins with adhesive properties (i.e. *COL1A1*, *COL3A1*, *COL4A6*, and *FBN1*), and inhibition of ECM degrading enzymes (i.e. MMP1; Fig. 6F). Using ELISA, dose dependent DHT regulation of Collagen 1 protein was confirmed (p<0.05; Fig. 6G). Significantly, in a set of human patient cancer-adjacent, BPH, and normal fibroblasts (CAF, BAF, and NPF respectively) we observed increased expression of *FBXO32* and *COL4A6* genes when treated with DHT in CAFs and BAFs only (p<0.05, Fig. 6H), and a marked decrease in expression of *MMP1* expression in all three cell types (p<0.05, Fig. 6I). Collectively, the above results suggest that stromal/fibroblast AR may act to alter the composition of the ECM, resulting in a pro-adhesive, anti-migratory matrix.

## DISCUSSION

Extensive analyses of cancerous epithelia have failed to significantly improve prediction of pre-existing prostate metastases or subsequent progression [44]. However, it has been known for over a decade that the level of stromal AR is inversely related to Gleason score, response to therapy, metastasis and subsequent biochemical relapse [25, 31-34]. This is the first study to associate decreased stromal AR levels with increased prostate cancer-related death, even in the context of older patients with significant disease burden at the time of diagnosis and initial management. Importantly, this now establishes that there is no maximum age at which stromal AR content cannot provide additional prognostic information. Conversely, since Gleason score in our cohort was found to be related to traditional tumor characteristics of poor prognosis, such as serum PSA, cancer-related death and epithelial AR content, the stromal AR results are likely reflective of what also happens in younger patients. In addition to confirming a protective role for stromal AR against prostate cancer progression, our data suggest that analysis of stromal AR levels and/or function may provide useful information regarding tumor aggressiveness and/or early metastasis, and could guide clinical decision making in younger and older men alike. This is particularly important in the latter group where there is a pervasive belief that older men are more likely to die with prostate cancer than from it.

Metastasis of solid tumors is accomplished by either proteolytic migration, involving secretion of ECM degrading enzymes to create space into which cells move, and/or amoeboid (non-proteolytic) squeezing of cells through the ECM. The amount and arrangement of ECM fibers, enzymes, and ECM pore size are capable of altering each type of migration, and have been implicated in malignant disease [45-47], and studies of malignant ovarian and breast cancers have identified defects in matrix protein cross-linking that render ECM more susceptible to proteolytic degradation [48, 49]. We show here that AR action in myofibroblasts leads to decreased expression of enzymes involved in ECM digestion and increased expression of key components of the ECM, both in our model cell line and primary patient fibroblasts. These results are supported by our findings that AR positive myofibroblasts produce a more adhesive ECM when treated with DHT, which inhibits migration and provides a less invasive environment for prostate cancer cells. Further work will be required to distinguish the role of androgen regulation of matrix degrading proteases. Collectively, our data suggest that fibroblast AR may play a key role in regulating cell attachment, and in organization of the ECM, and that a loss of stromal AR creates a passive ECM environment that is less adhesive for cancer epithelia and more conducive for metastatic spread (Fig. 6H). We predict that defining the precise contribution that AR makes to ECM composition may inform on early disease spread and therefore overall patient outcome.

It appears from our results and those of others that stromal AR may also promote prostate cancer proliferation, as suggested here by the production of an unidentified soluble mediator, and/or ECM-bound growth factor [50-52]. On the surface, this appears at odds with clinical data demonstrating an association between low stromal AR and death from prostate cancer. Given decreased stromal AR expression throughout progression however [13, 50, 53, 54], or as shown here with increasing Gleason score, these two findings may not be as paradoxical as might be thought. Indeed, stromal AR may be pro-proliferative in early prostate cancer; exogenous tumors in mice grow larger when associated with AR sensitive stroma [55], and are inhibited by stroma lacking AR [50]. Conversely *in vivo* knockdown of stromal AR was found to be more effective at inhibiting tumor growth in early stages of progression rather than at later stages [50, 56]. In this study, there was no difference between take rate or cellular morphology of human tumors grafted with either AR positive or AR negative myofibroblasts. Instead, we found in grafts containing AR positive myofibroblasts that cancer cells exhibit increased apoptosis following castration. Collectively, these findings suggest that stromal AR can play a pro-proliferative, pro-adhesive and/or anti-migratory role in prostate cancer. It is entirely possible that stromal AR is pro-tumorigenic in very early stage disease, but prevents metastasis of evolving epithelial cancer cells by altering the composition and permissiveness of the ECM.

In conclusion, this manuscript is the first to show that unique androgen/AR transcriptional responses in prostate myofibroblasts play an important role in stromal-mediated alterations to the ECM and microenvironment. Clinically, it will be important to determine the key factors affected by a loss of stromal AR that may influence patient outcome and could be exploited by targeted therapies. The precise composition of the ECM may be one such key mediator of epithelial cancer cell invasiveness and thus indicative of patient outcome, tumor aggressiveness and treatment response.

## MATERIALS AND METHODS

### Clinical cohorts

The South Australia Prostate Cancer Clinical Outcomes Collaborative (SA-PCCOC; http://www.sa-pccoc.com/) tracks men diagnosed with prostate cancer in the South Australian public health system. Using the SA-PCCOC database, we identified 66 sequential patients whom underwent TURP for symptomatic relief of BPH urinary obstruction at the Repatriation General Hospital (RGH; Daws Park, South Australia) between 2000 and 2007, in which there was (i) a first diagnosis of prostate cancer on histological Gleason grading, (ii) cancer comprising >5% of the specimen, and (iii) sufficient areas of BPH and cancer in each sample from which multiple cores could be obtained. Areas of BPH and cancer were identified by H&E staining and mapped onto paraffin embedded material by a pathologist. Duplicate five mm cores of BPH and cancer from each individual were then used to generate tissue microarrays. Clinical data relating to each patient was acquired from the SA-PCCOC database. Sample and data acquisition was performed according to protocols approved by the Flinders Medical Centre and RGH Ethics Committees (Protocol #042/10).

Immunohistochemistry was performed with the AR N-20 antisera (Santa Cruz Biotechnology) and, detected using the LSAB+ System-HRP kit (Dako Laboratories, CA, USA). Staining was scored additively by two researchers in three independent fields from 0 (no staining) to 3 (very intense staining), yielding sample scores of 0-9 in epithelial and stromal compartments of both cancer and BPH. No stromal compartment achieved very intense staining. The mean sample score from the two researchers yielded the AR staining intensity score. Differences in staining intensity, Gleason Score, serum PSA and percent prostate cancer were assessed using two-tailed Mann-Whitney U tests. In samples dichotomized by median AR level, differences in prostate cancer-specific death were assessed using Barnard's Exact test. Significance was set at p<0.05.

Human tissue was obtained from consented patients in accordance with Human Ethics Research Approvals 34306 at Epworth Hospital, 03-14-04-08 at Cabrini Hospital and RMO 2006/61082004000145 at Monash University, and processed as previously published [18]. Briefly, tissue from patients with BPH or Gleason score 7 prostate cancers were extracted from TURP and radical prostatectomy specimens respectively. Primary fibroblasts, representing CAFs, BAFs and NPFs were isolated from patient specimens, cultured in RPMI with 5% FCS and 100nM testosterone or equivalent vehicle (ethanol), and assessed *in vitro* between passages 3-6. The integrity of primary fibroblast cultures was confirmed *in vitro* by growth properties, immunological markers and RNA expression, and their tumorigenic potential in vivo using tissue recombination with BPH-1 cells.

### Cell lines

For *in vitro* experiments C4-2B [57] and PC-3 (ATCC, VA, USA) prostate cancer epithelial cells, telomerase immortalized human prostate stromal myofibroblast cells expressing AR (PshTertAR) or matched empty vector control (PShTert-ctrl) [31], and WMPY human prostate fibroblasts expressing Hic-5 or scrambled shRNA [58] were used. All cell lines were authenticated via Short Tandem Repeat testing in 2014, completed at CellBank Australia (NSW, Australia). In experimental conditions cells were incubated in stripped medium (Phenol Red Free-RPMI 1640 with 5% dextran coated (DCC) FBS) supplemented with 10 nM DHT or vehicle, or 10 μM bicalutamide (BIC). For conditioned media, confluent PShTert-AR and PShTert-ctrl cells were incubated in stripped medium (Phenol Red Free-RPMI 1640 with 5% dextran coated (DCC) FBS) supplemented with 10 nM DHT or vehicle. Media was collected at 6, 12, 18, 24, 36, or 48 h after initial treatment, centrifuged to remove debris, filtered and frozen, and subsequently used neat for transactivation assays or at a 1:1 dilution with fresh stripped media for other cell studies.

### Transactivation assays

Transactivation studies were performed as described previously [59] using Lipofectamine 2000^™^ (Life Technologies, CA, USA) or LTX-plus (Life Technologies) for transfection of luciferase constructs. Following transfection, cells were treated with 0.1-1000 nM of steroids or equivalent vehicle (ethanol) control for 22 h. Results are presented as mean (± SEM) of six independently transfected wells.

### Chromatin immunoprecipitation (ChIP)

ChIP was performed as described previously [59], using semi-confluent PShTert-AR or C4-2B cells were treated for 4 hours with 10nM DHT or vehicle. Cells were then formaldehyde fixed and sonicated to produce 300-1500 bp fragments. Lysates were pre-cleared with yeast tRNA and protein G sepharose, and immunoprecipitated overnight with 4 μg of AR N-20 (Santa Cruz Biotechnology) or rabbit IgG (Santa Cruz Biotechnology) antiserum. Protein-DNA complexes were eluted from the beads, digested with proteinase K and was DNA purified by phenol-chloroform extraction. Resulting DNA samples were assessed by RT-qPCR in triplicate, with primers listed in Supplementary Table 1. Data was calculated as percent input and normalized to non-specific control (NC2). Results are representative of three independent experiments.

### ELISA

ELISA was used to measure collagen 1 levels in media collected from confluent PShTert-AR myofibroblasts treated with 50 μg/ml ascorbic acid (Sigma-Aldrich, NSW, Australia) and either DHT, vehicle control and or BIC. Media collected from six independent treated confluent cells was plated into 96-well Maxisorp (Nunc, Simga Aldrich) plates and incubated overnight at 4ÐC. Plates were washed in PBS supplemented with 0.1% Tween (PBST), blocked in 2.5% BSA and washed in PBST, plates were probed with rabbit anti-collagen type 1 antibody (0.25 μg/ml, Rockland Immunochemistry, PA, USA) for 3 h and detected via a europium-tagged anti-rabbit secondary antibody. The concentration of collagen was subsequently fluorescently detected using 340 nm excitation/615 nm emission spectra.

### Tissue recombination

Renal capsule tissue recombination grafting of PShTert-AR or PShTert-ctrl cells with pieces of patient-derived primary human prostate cancer tissue into NOD-SCID mice was performed and analyzed as previously described [18, 35, 60]. Briefly, PShTert-AR or PShTert-ctrl cells (2.5 × 10^5^) were combined with 2 mm X 2 mm X 1 mm pieces of patient-derived primary human prostate cancer tissue in 30 μl of collagen/RPMI 1640 + 5% FBS with 0.1% penicillin-streptomycin for 24 h, and grafted under the renal capsule of NOD-SCID mice for 8 weeks. Mice were castrated, and grafts allowed to grow for an additional 3 days before being removed, paraffin-embedded and sectioned. Immunohistochemistry for Ki-67 (Sigma-Aldrich), caspase-3 (Sigma-Aldrich), and AR (Sigma-Aldrich) was performed.

### Microarrays

RNA extracted from cells treated with either DHT or vehicle using the RNeasy Kit (Qiagen, Melbourne, Australia), was analyzed using Affymetrix 1.0st Gene Arrays. Data was Bioinformatically analyzed using either in R using Gene Ontology categories, or in R or using DAVID Bioinformatics Resources http://david.abcc.ncifcrf.gov/home.jsp (46, 47).

### Quantitative real-time PCR (RT-qPCR)

cDNA created from sample RNA was analyzed via RT-qPCR as previously described [61], using SYBR Green (Biorad) and primer pairs detailed in Supplementary Table 1. Data is presented relative to *GAPDH, PPIA*, and *mRPL19* as per GeNorm (http://medgen.ugent.be/~jvdesomp/genorm/#introduction).

### Immunoblot

Protein lysates in RIPA buffer were prepared as previously described [59] and immunostained with anti-AR (N20, Santa Cruz Biotechnology), anti-FKBP5 (H100, Santa Cruz Biotechnology), anti-alpha tubulin (05-829, Millipore, Bedford, MA), or anti-β-actin (A1978, Sigma-Aldrich).

### Proliferation, adhesion and motility

Proliferative response of PShTert-AR or C4-2B cells to DHT and or BIC was measured in quadruplicate via Trypan blue exclusion. Adhesion of PShTert-AR, PShTert-ctrl, or C4-2B cells was measured using an adhesion assay as described previously [62]. Briefly, 5 × 10^4^ PShTert-AR, PShTert-ctrl, or C4-2B cells were added to 24-well plates containing treatment media and left to adhere for 15-240 min at 37ÐC. Media was removed and cells were washed with PBS before manual counting. Cellular attachment (trypsinization resistance) was measured using a crystal violet assay adapted from a previous study [62]. Briefly, PShTert-AR or C4-2B cells were plated in stripped media (5 × 10^4^ cells/well in 96 well plates) overnight and treated with 1-100 nM DHT ± 10 μM BIC or equivalent vehicle control for 16 h. Cells were washed with PBS and incubated with trypsin for 2.5 - 15 min. Cells were washed, ethanol fixed and stained with 1% crystal violet solution. Dye was eluted from cells with 10% glacial acetic acid and the concentration measured at an absorbance of 595 nm. Motility and invasion was tested described previously [63], using calcein labelled C4-2B cells were applied to modified Boyden chambers (ChemoTx, Neuro Probe). Calcein AM was measured in the bottom wells using a FLUOstar OPTIMA plate reader at 480 nm excitation and 520 nm emission wavelengths.

### Conditioned matrix

Matrices produced from confluent fibroblasts treated with 50 μg/ml ascorbic acid and 10 nM DHT or vehicle or 10μM BIC, were decellularized with EDTA and used in adhesion assays (above) and trypsinization assays adapted from previous descriptions [62].

### 3D-matrices

3D-matrices were produced from DHT or vehicle treated fibroblasts seeded into gelatin coated wells as previously described [43]. After decellularization with extraction buffer containing PBS, 0.28% ammonium hydroxide (Sigma-Aldrich), and 0.5% Triton-X (Sigma-Aldrich), the remaining 3D-matrix was used for adherence, proliferation, invasion, and motility/gap closure assays.

When the cells had grown to 100% confluence, media was replaced with stripped media supplemented with 50 μg/ml ascorbic acid and 10 nM DHT or equivalent vehicle control. Treatment was repeated every 48 h. After 8 days, myofibroblasts were removed via an extraction buffer containing PBS, 0.28% ammonium hydroxide (Sigma-Aldrich), and 0.5% Triton-X (Sigma-Aldrich). Remaining 3D matrix was gently washed in PBS prior to adherence, proliferation and invasion assays.

For cancer cell gap closure assays, into each well, sterile silicon culture-inserts (Ibidi 80209) were positioned into wells containing 3-D matrices, and PC3 cells (3.5 × 10^4^ cells per chamber) in stripped medium were aliquoted. Following 16h by incubation, Ibidi inserts were removed, leaving a 500μm cell-free gap. Migration of PC3 cells across the gap was monitored for 0, 3, 7, 11, and 15 h time-points, using a Zeiss Axio Observer.Z1 with HBO 100 microscope illuminating system (Zeiss, Göttingen, Germany). Migration was measuring as cell-free gap-closure using AxioVision Rel 4.8 software, and analysed with the MRI Wound Healing Tool (ImageJ software, version 1.47v).

## SUPPLEMENTAL MATERIAL TABLES AND FIGURES


