# Erratum: Stromal androgen receptor regulates the composition of the microenvironment to influence prostate cancer outcome

**DOI:** 10.18632/oncotarget.6263

**Published:** 2015-11-02

**Authors:** Damien A. Leach, Eleanor F. Need, Roxanne Toivanen, Andrew P. Trotta, Helen M. Palethorpe, David J. Tamblyn, Tina Kopsaftis, Georgina M. England, Eric Smith, Paul A. Drew, Carole B. Pinnock, Peng Lee, Jeff Holst, Gail P. Risbridger, Samarth Chopra, Donald B. DeFranco, Renea A. Taylor, Grant Buchanan

Oncotarget. 2015; 6:16135-16150

***PMID:*** *25965833*

The author name is incorrect.

Helen M. Pale**n**thorpe

The corrected author name is provided here.

Helen M. Palethorpe

