# Supplementary material for: Stromal androgen receptor regulates the composition of the microenvironment to influence prostate cancer outcome
Source: Oncotarget. 2015 Apr 19;6(18):16135–50. doi: 10.18632/oncotarget.3873 (PMC4599261; doi:10.18632/oncotarget.3873)
Supplement: Supplementary file 1 [file oncotarget-06-16135-s001.pdf]

Stromal androgen receptor regulates the composition of the microenvironment to influence prostate cancer outcome

Supplementary Material

Supplementary Table 1: PCR Primers

Use[@]	Gene[%]	Direction	Sequence*	Refs[#]
Q	GAPDH	forward	GTCATGGGTGTGAACCATGAGA	
		reverse	GGTCATGAGTCCTTCCACGATAC	(11)
Q	PSA	forward	GGCAGCATTGAACCAGAGGAG	
		reverse	GCATGAACTTGGTCACCTTCTG	(11)
Q	FKBP5	forward	ATTATCCGGAGAACCAAACG	
		reverse	CAAACATCCTTCCACCACAG	
Q	ABCC4	forward	CCCCGTGGGAGCAGGGAAGT	
		reverse	CCGAGAACACCCAGGGCTGC	
Q	WNT5A	forward	AAGGAGTTCGTGGACGCCCG	
		reverse	GCAGGCCACATCAGCCAGGT	
Q	SLC45A3	forward	GCCTCCCTCTACCACCGGGA	
		reverse	GCCTGGCAGGAAGCTGGTCA	
Q	NKX3-1	forward	CCGAGACGCTGGCAGAGACC	
		reverse	GTGGGAGAAGGCAGCTCGGG	
Q	FBXO32	forward	CCCTTCAGCTCTGCAAACACTGTC	
		reverse	CTCCAGTCAGCAGGGGGACC	
Q	TGFB3	forward	GGCCCTTGCCCATACCTCCG	
		reverse	AGCAAGGCGAGGCAGATGCT	
Q	FGF5	forward	CGGATGGCAAAGTCAATGGATCC	
		reverse	CGCTCCCTGAACTTGCAGTCAT	
Q	PPIA	forward	GCATACGGGTCCTGGCAT	
		reverse	ACATGCTTGCCATCCAACC	
Q	MRPL19	forward	TGCCAGTGGAAAAATCAGCCA	
		reverse	CAAAGCAAATCTCGACACCTTG	
Q	FBN1	forward	CTCCTGGAAGTTTTGTCTGTACCTGC	
		reverse	GGGCTGTTCTTGCAGACTCCATTA	
Q	COL1A1	forward	AGGGCTCCAACGAGATCGAGATCCG	
		reverse	TACAGGAAGCAGACAGGGCCAACGTCG	
Q	COL3A1	forward	AGCTGGCTACTTCTCGCTCTGCTT	
		reverse	CGCATAGGACTGACCAAGATGGG	
Q	COL4A6	forward	AGGACTGCAGTGGGAGCTGTCAGT	
		reverse	AGGACCTGTTGGGCCTTGAATTC	
Q	MMP1	forward	GACGTTCCCAAAATCCTGTCCAG	
		reverse	GGTAGAAGGGATTTGTGCGCATGT	
C	NC2	forward	GTGAGTGCCCAGTTAGAGCATCTA	
		reverse	GGAACCAGTGGGTCTTGAAGTG	(12)
C	FKBP5	forward	GCTCTGACTTATTGTTCTCTTACTGCCC	
		reverse	TTGCTGTCAGCACATCGAGTTCA	(13)
C	PSA	forward	GCCTGGATCTGAGAGAGATATCATC	
		reverse	ACACCTTTTTTTTTCTGGATTGTTG	(11)
C	FBXO32	forward	GGCTCTCCAGCCGTGCATGA	
		reverse	AGCAGGTGTGCACGTCCCTC	

@ Primers used in either RT-QPCR (Q) or ChIP (C)
% Gene primer raised against
* Sequence primer raised against
# Reference for primers used, were applicable

Supplementary Table 2: Androgens enrich different cell functional pathways in fibroblasts compared to epithelial cells

Category name@	PshTERT-AR					C4-2B				
	Fold Enrichment%		p value#	No. of Genes*	examples	Fold Enrichment%		p value#	No. of Genes*	examples
GO:0007155~cell adhesion	**+**	**1.83**	**0.004**	**36**	**CLDN7, ARHGAP6, PTK2B, LYVE1**	NR	-	-	0	-
GO:0007156~homophilic cell adhesion	**+**	**3.39**	**0.5E-04**	**9**	**PCDHB8, CADM1, PCDHB2, PCDH9**	-	1.78	0.30	5	PCDHB2, CELSR1, CDH3, CDH6
GO:0005578~proteinaceous extracellular matrix	**+**	**3.06**	**5.0E-05**	**19**	**LOX, WNT5A, LUM, COL3A1**	NR	0.00	-	0	-
GO:0005583~fibrillar collagen	**+**	**1.06**	**0.03**	**3**	**LUM, COL3A1, COL5A2**	NR	0.00	-	0	-
GO:0032963~collagen metabolic process	**+**	**5.80**	**0.03**	**4**	**HIF1A, COL3A1, ADAMTS3, ADAMTS2**	NR	0.00	-	0	-
GO:0042981~regulation of apoptosis	**+**	**1.78**	**0.05**	**29**	**IER3, TRAIP, BIRC5, TGFB3**	**-**	**1.63**	**0.0128**	**28**	**BLACF1, NFKB1, BARD1, TP53INP1**
GO:0001568~blood vessel development	-	0.91	0.736	6	ARHGAP22, VEGFC, MYOCD, JUN	**+**	**2.18**	**0.0125**	**14**	**VEGFA, THBS1, ANGPT2, ZMIZ1**
GO:0022403~cell cycle phase	**-**	**8.35**	**7.82E-75**	**96**	**CDKN1A, PPP3CA, CCNG1, PPP1CB**	NR	-	-	0	-
GO:0000278~mitotic cell cycle	**-**	**7.80**	**9.31E-39**	**96**	**CDKN1A, PPP3CA, CCNG1, PPP1CB**	NR	-	-	0	-
GO:0040017~positive regulation of locomotion	**-**	**2.45**	**0.044**	**8**	**PLD1, PDGFRB, THBS1, SCG2**	**+**	**3.8**	**0.01**	**10**	**IL8, JUB, LAMB1, ITGA**
GO:0040017~positive regulation of cell motion	**-**	**2.45**	**0.04**	**8**	**PTK2B, BCL6, JAK2, THBS1**	**+**	**1.8**	**0.01**	**10**	**LYN, JUB, LAMB1, ITGA**
GO:0051726~regulation of cell cycle	**-**	**4.90**	**4.35E-22**	**54**	**KNTC1, MYC, CDK1, JUN**	NR	-	-	0	-
GO:0000087~M phase of mitotic cell cycle	**-**	**13.52**	**6.18E-68**	**79**	**CDK2, KIF2C, NCAPD3, CDCA8**	+	0.68	0.94	4	MPHOSPH9, MAP9, CDC26, NCAPD3
GO:0010740~positive regulation of protein kinase cascade	NR	0.00	-	0	-	**+**	**3.65**	**0.00003**	**16**	**HIPK2, TICAM2, TGM2, KCNRG**
GO:0009967~positive regulation of signal transduction	NR	0.00	-	0	-	**+**	**2.58**	**0.0003**	**20**	**GOLT1B, TAOK3, PCK2, HIPK2**

@ Functional pathway analysis of the top up and down genes regulated by androgen in C4-2B and PShTert-AR cells.
% Fold change in functional pathway enrichment or depletion
* Number of regulated genes in each category out of the 1000 genes initially inputted from each cell type
# Modified Fisher Exact P-Value score
NR = not regulated. +/- represents direction of regulation

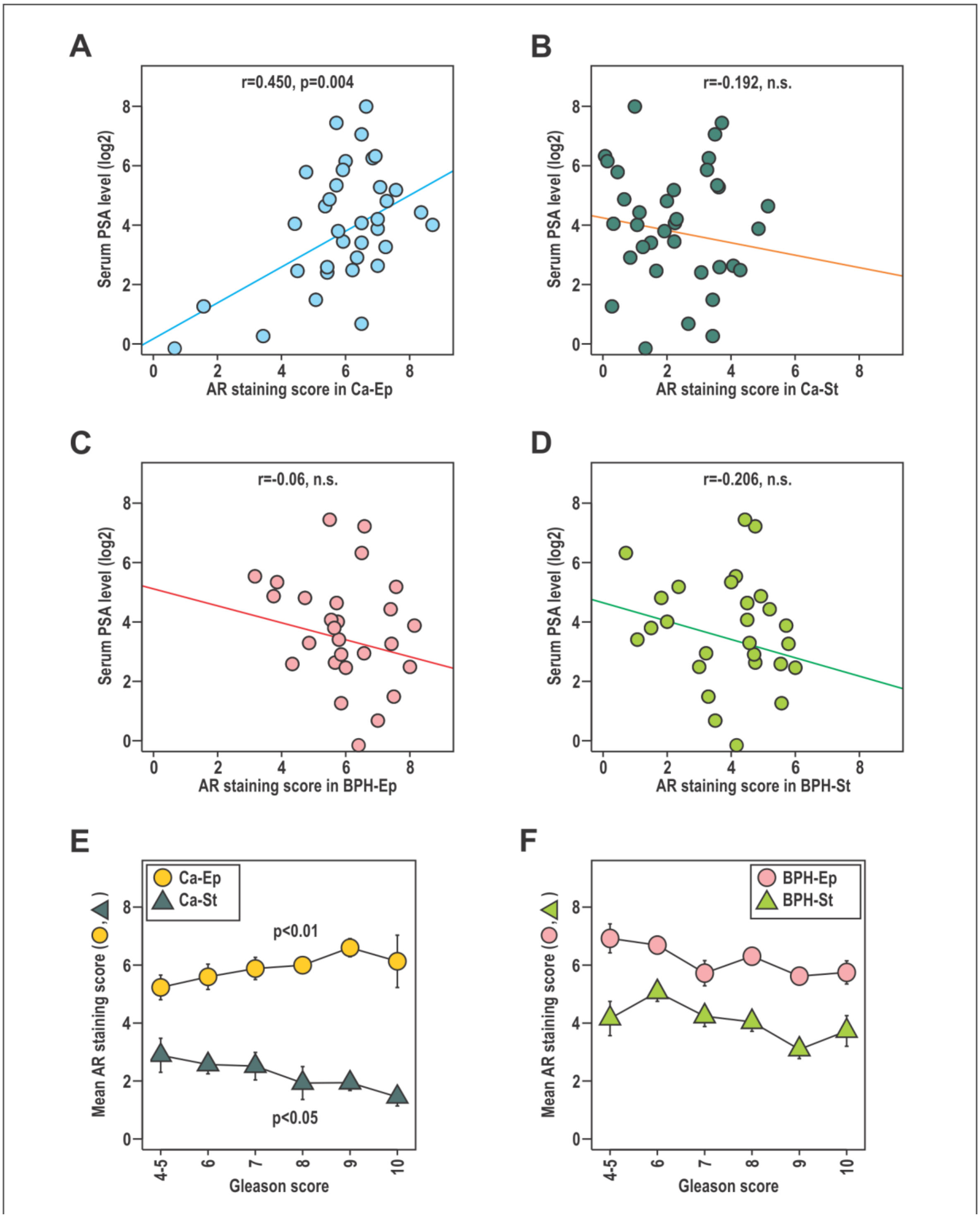

Supplementary Figure S1: Stromal and epithelial AR in relation to Gleason grade and serum PSA. A-D. The average AR score from each compartment in benign or cancerous state were analyzed in relation to serum PSA. **E.** Mean AR scores for stromal (St) and epithelial (Ep) compartments in patient BPH samples (stained overnight with anti-AR N20) were analyzed in relation to Gleason grade of the matched cancer sample.

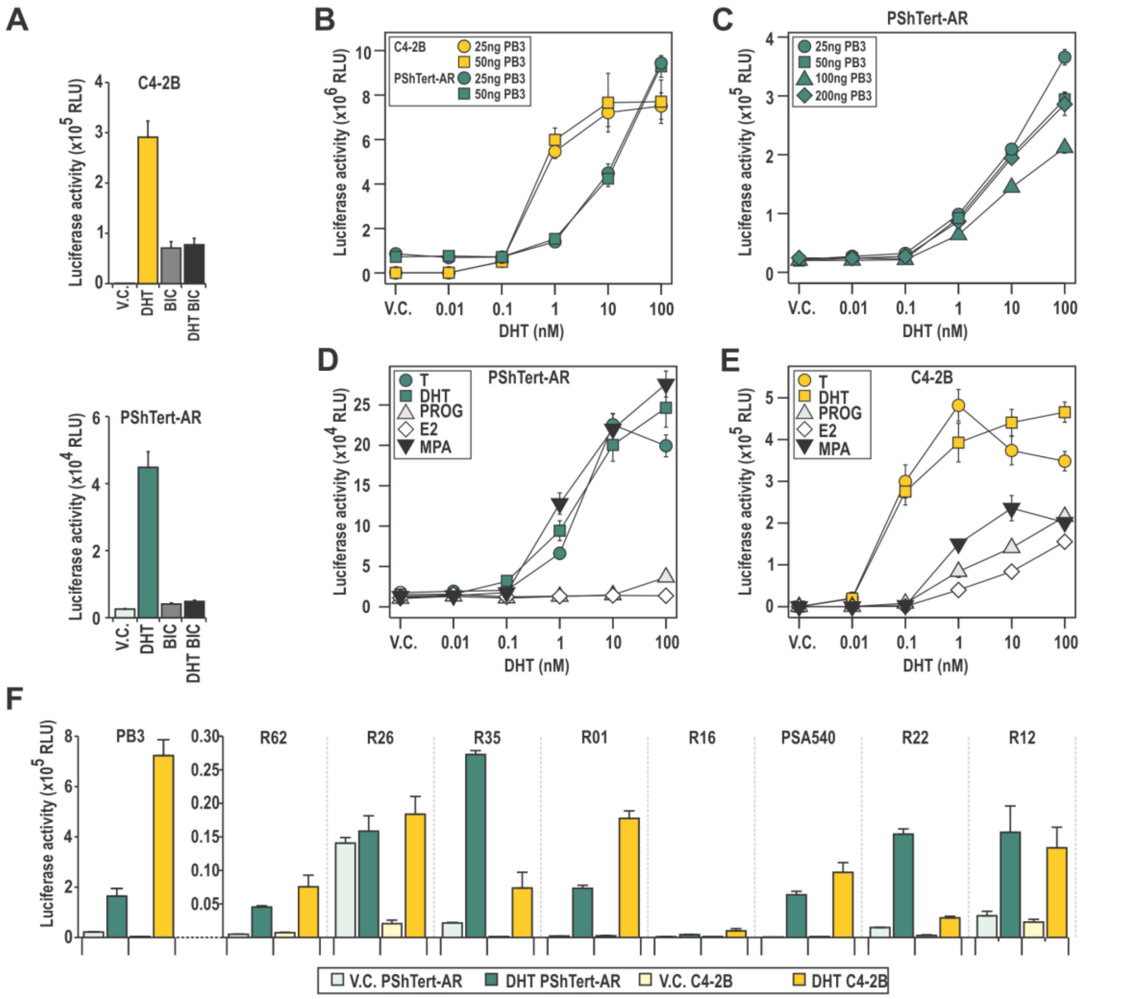

Supplementary Figure S2: Fibroblast and epithelial androgen signaling in response to reporter concentration and different ligands. A. C4-2B and PShTert-AR cells were transfected with probasin reporter (PB3) and treated with 10 nM vehicle or DHT in the presence or absence of bicalutamide (BIC). **B-C**. C4-2B and PShTert-AR cells were transfected with 25-200 ng of PB3 reporter vector and treated with 0-100 nM DHT as described in materials and methods. Data represents mean ± SEM of six independently transfected wells. **D,E**. PShTert-AR (**D**) and C4-2B (**E**) cells were transfected with 25 ng probasin reporter (PB3) and treated with 0-100 nM testosterone (T), dihydrotestosterone (DHT), progesterone (PROG), estradiol (E2), and medroxyprogesterone acetate (MPA). **F**. C4-2B and PShTert-AR cells were transfected with a variety of AR reporter constructs as described in (4, 12, 14), and stimulated with 10 nM DHT. Data represents mean ± SEM relative light units (RLU) of six independently transfected wells.

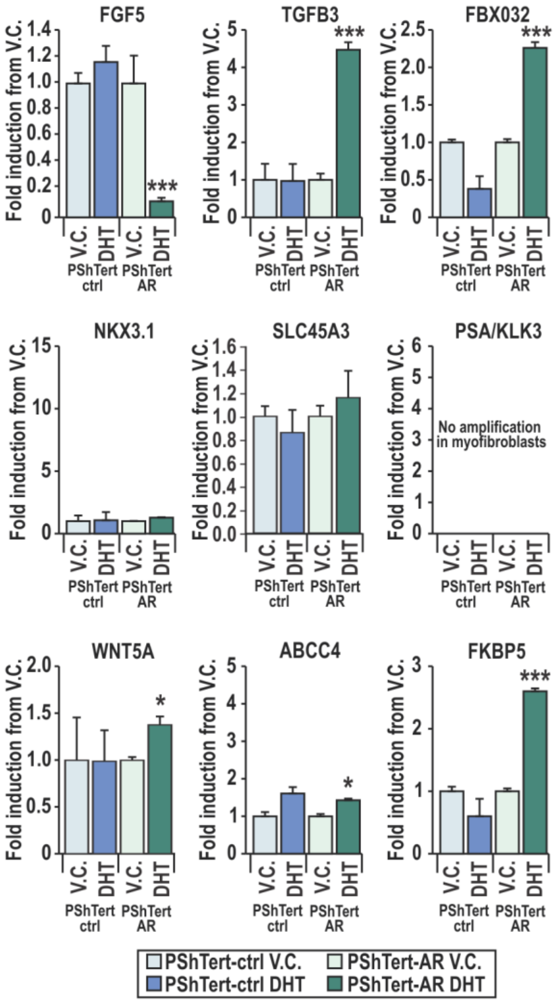

Supplementary Figure S3: Microarray validation. Triplicate RNA samples from 10 nM DHT or vehicle treated PShTert-ctrl or PShTert-AR cells were pooled and analyzed via RT-qPCR. Data represents mean + SEM of triplicate biological replicates measured in duplicate PCR samples. Significance between DHT and V.C. treatments was calculated via Student's T-test; * $p<0.05$, ** $p<0.01$, ***$p<0.001$.

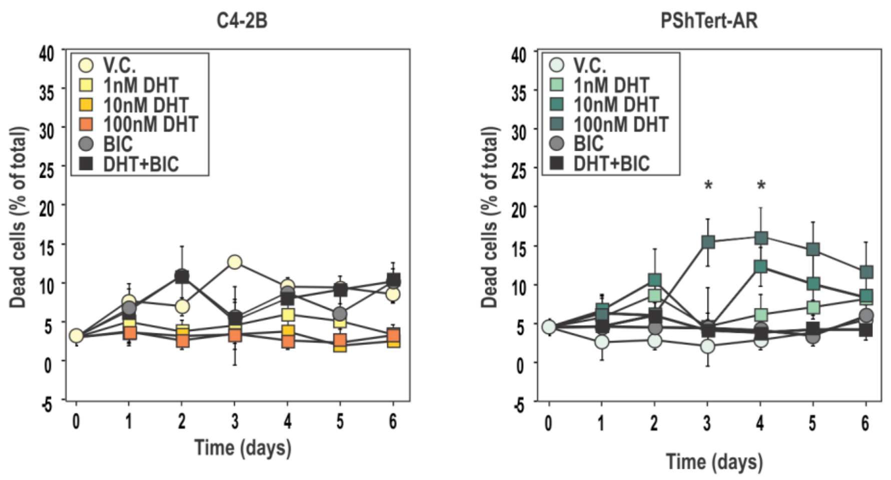

Supplementary Figure S4: Cell death in response to androgen treatment.

PShTert-AR or C4-2B cells (1.5 × 10^3 cells/well in 24 well plates) were treated with 0.1-100 nM DHT, and/or 10 µM bicalutamide (BIC), or equivalent vehicle control. Dead cells were analyzed as previously described (15), and are presented as the mean ± SEM of the percentage of total cells.

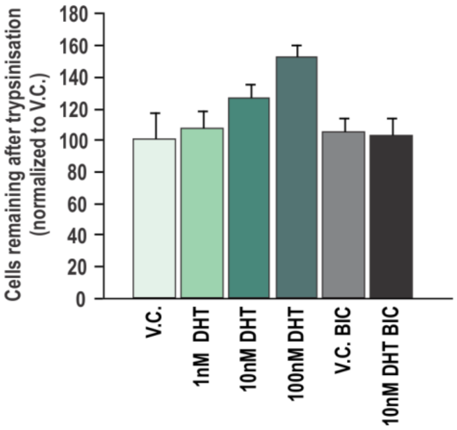

Supplementary Figure S5: PShTert-AR cells (1 × 10^4 cells/well in 96 well plates) were treated with 0.1-100 nM DHT, and/or 10 µM bicalutamide (BIC), or equivalent vehicle control. Cells were treated with trypsin for 5 minutes, and remaining attached cells were stained with crystal violet. Data represents mean ± SEM absorbance of six independently transfected wells and is presented as the percentage of vehicle control.

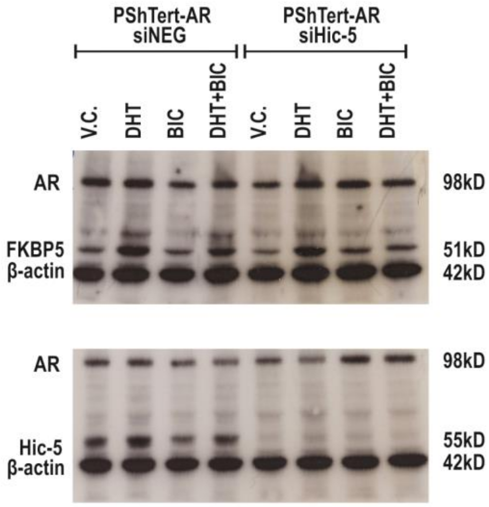

Supplementary Figure S6: Hic-5 silencing alters Hic-5 protein levels but does not affect AR transactivation of FKBP5 Lysates from PShTert-AR cells transfected with siRNA against Hic-5 or control siRNA were treated with 10 nM DHT or equivalent vehicle control (V.C.) and 10 µM bicalutamide (BIC). Lysates were prepared as described in materials and methods, and were probed using anti-AR N-20 (Santa Cruz Biotehnology), anti-FKBP5 H100 (Santa Cruz Biotehnology), and anti-Hic-5 611165 (BD Transduction Laboratories, USA). Anti-β-Actin (Millipore, Bedford, MA) was used as a loading control.

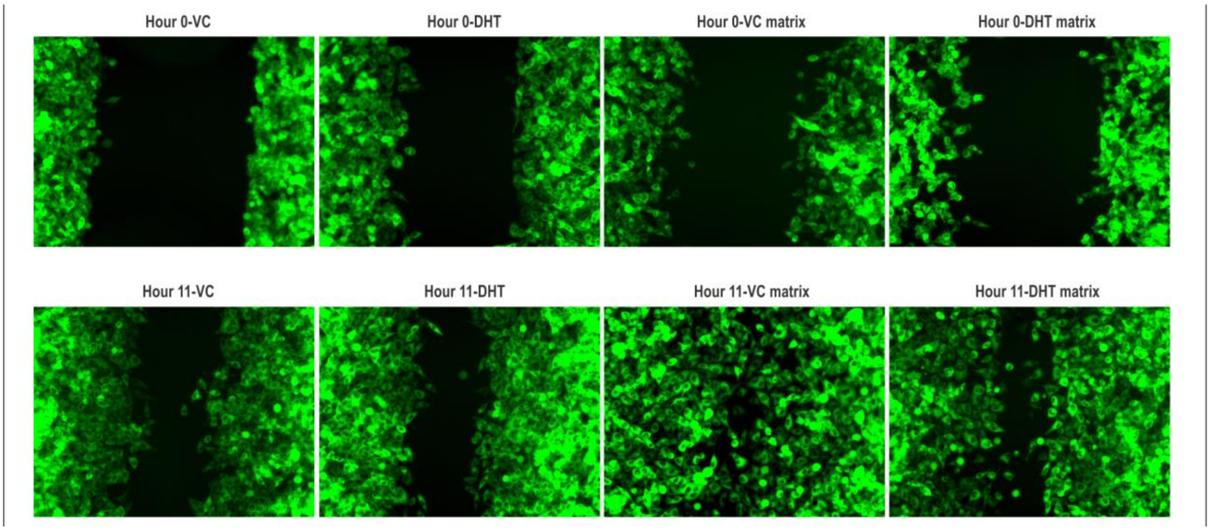

Supplementary Figure S7: PC-3 gap closure across PShTert-AR derived matrix. PC-3 cells expressing GFP were seeded onto matrices created by PShTert-AR treated with or without DHT, or non-matrix controls. Using an Ibidi chamber a 500um space was created in the PC-3 monolayer. The closure of this gap was measured over a 15 hour period.

References citied

1. Wu HC, Hsieh JT, Gleave ME, Brown NM, Pathak S, Chung LW. Derivation of androgen-independent human LNCaP prostatic cancer cell sublines: role of bone stromal cells. Int J Cancer. 1994 May 1;57(3):406-12. PubMed PMID: 8169003. Epub 1994/05/01. eng.
2. Li Y, Li CX, Ye H, Chen F, Melamed J, Peng Y, et al. Decrease in stromal androgen receptor associates with androgen-independent disease and promotes prostate cancer cell proliferation and invasion. J Cell Mol Med. 2008 Dec;12(6B):2790-8. PubMed PMID: 18266956. Epub 2008/02/13. eng.
3. Heitzer MD, DeFranco DB. Mechanism of action of Hic-5/androgen receptor activator 55, a LIM domain-containing nuclear receptor coactivator. Mol Endocrinol. 2006 Jan;20(1):56-64. PubMed PMID: 16141357. Epub 2005/09/06. eng.
4. Need EF, Scher HI, Peters AA, Moore NL, Cheong A, Ryan CJ, et al. A novel androgen receptor amino terminal region reveals two classes of amino/carboxyl interaction-deficient variants with divergent capacity to activate responsive sites in chromatin. Endocrinology. 2009 Jun;150(6):2674-82. PubMed PMID: 19282387. Pubmed Central PMCID: 2689802. Epub 2009/03/14. eng.
5. Taylor RA, Toivanen R, Frydenberg M, Pedersen J, Harewood L, Australian Prostate Cancer B, et al. Human epithelial basal cells are cells of origin of prostate cancer,

independent of CD133 status. Stem Cells. 2012 Jun;30(6):1087-96. PubMed PMID: 22593016. Epub 2012/05/18. eng.
6. Lawrence MG, Taylor RA, Toivanen R, Pedersen J, Norden S, Pook DW, et al. A preclinical xenograft model of prostate cancer using human tumors. Nat Protoc. 2013 Apr 4;8(5):836-48. PubMed PMID: 23558784. Epub 2013/04/06. eng.
7. Toivanen R, Berman DM, Wang H, Pedersen J, Frydenberg M, Meeker AK, et al. Brief report: a bioassay to identify primary human prostate cancer repopulating cells. Stem Cells. 2011 Aug;29(8):1310-4. PubMed PMID: 21674698. Epub 2011/06/16. eng.
8. Trotta AP, Need EF, Butler LM, Selth LA, O'Loughlin MA, Coetzee GA, et al. Subdomain structure of the co-chaperone SGTA and activity of its androgen receptor client. J Mol Endocrinol. 2012 Oct;49(2):57-68. PubMed PMID: 22693264. Epub 2012/06/14. eng.
9. Humphries MJ. Cell adhesion assays. Methods Mol Biol. 2009;522:203-10. PubMed PMID: 19247616. Epub 2009/02/28. eng.
10. Castello-Cros R, Cukierman E. Stromagenesis during tumorigenesis: characterization of tumor-associated fibroblasts and stroma-derived 3D matrices. Methods Mol Biol. 2009;522:275-305. PubMed PMID: 19247611. Pubmed Central PMCID: 2670062. Epub 2009/02/28. eng.
11. Jia L, Kim J, Shen H, Clark PE, Tilley WD, Coetzee GA. Androgen receptor activity at the prostate specific antigen locus: steroidal and non-steroidal mechanisms. Mol Cancer Res. 2003 Mar;1(5):385-92. PubMed PMID: 12651911. Epub 2003/03/26. eng.
12. Jia L, Berman BP, Jariwala U, Yan X, Cogan JP, Walters A, et al. Genomic androgen receptor-occupied regions with different functions, defined by histone acetylation, coregulators and transcriptional capacity. PLoS One. 2008;3(11):e3645. PubMed PMID: 18997859. Pubmed Central PMCID: 2577007. Epub 2008/11/11. eng.
13. Denny WB, Prapapanich V, Smith DF, Scammell JG. Structure-function analysis of squirrel monkey FK506-binding protein 51, a potent inhibitor of glucocorticoid receptor activity. Endocrinology. 2005 Jul;146(7):3194-201. PubMed PMID: 15802496. Epub 2005/04/02. eng.
14. Buchanan G, Ricciardelli C, Harris JM, Prescott J, Yu ZC, Jia L, et al. Control of androgen receptor signaling in prostate cancer by the cochaperone small glutamine rich tetratricopeptide repeat containing protein alpha. Cancer Res. 2007 Oct 15;67(20):10087-96. PubMed PMID: 17942943. Epub 2007/10/19. eng.
15. Marrocco DL, Tilley WD, Bianco-Miotto T, Evdokiou A, Scher HI, Rifkind RA, et al. Suberoylanilide hydroxamic acid (vorinostat) represses androgen receptor expression and acts synergistically with an androgen receptor antagonist to inhibit prostate cancer cell proliferation. Mol Cancer Ther. 2007 Jan;6(1):51-60. PubMed PMID: 17218635. Epub 2007/01/16. eng.
